# Modeling the Winter–to–Summer Transition of Prokaryotic and Viral Abundance in the Arctic Ocean

**DOI:** 10.1371/journal.pone.0052794

**Published:** 2012-12-20

**Authors:** Christian Winter, Jérôme P. Payet, Curtis A. Suttle

**Affiliations:** 1 Department of Marine Biology, University of Vienna, Vienna, Austria; 2 Department of Microbiology, Oregon State University, Corvallis, Oregon, United States of America; 3 Departments of Earth, Ocean and Atmospheric Sciences, Microbiology and Immunology, Botany, and the Biodiversity Research Centre, The University of British Columbia, Vancouver, British Columbia, Canada; Uppsala University, Sweden

## Abstract

One of the challenges in oceanography is to understand the influence of environmental factors on the abundances of prokaryotes and viruses. Generally, conventional statistical methods resolve trends well, but more complex relationships are difficult to explore. In such cases, Artificial Neural Networks (ANNs) offer an alternative way for data analysis. Here, we developed ANN-based models of prokaryotic and viral abundances in the Arctic Ocean. The models were used to identify the best predictors for prokaryotic and viral abundances including cytometrically-distinguishable populations of prokaryotes (high and low nucleic acid cells) and viruses (high- and low-fluorescent viruses) among salinity, temperature, depth, day length, and the concentration of Chlorophyll-*a*. The best performing ANNs to model the abundances of high and low nucleic acid cells used temperature and Chl-*a* as input parameters, while the abundances of high- and low-fluorescent viruses used depth, Chl-*a*, and day length as input parameters. Decreasing viral abundance with increasing depth and decreasing system productivity was captured well by the ANNs. Despite identifying the same predictors for the two populations of prokaryotes and viruses, respectively, the structure of the best performing ANNs differed between high and low nucleic acid cells and between high- and low-fluorescent viruses. Also, the two prokaryotic and viral groups responded differently to changes in the predictor parameters; hence, the cytometric distinction between these populations is ecologically relevant. The models imply that temperature is the main factor explaining most of the variation in the abundances of high nucleic acid cells and total prokaryotes and that the mechanisms governing the reaction to changes in the environment are distinctly different among the prokaryotic and viral populations.

## Introduction

The Arctic Ocean is characterized by environmental extremes and is subject to large seasonal differences in ice cover, availability of sunlight, and river discharge that collectively set the pace for marine life in the area. This polar region is influenced by the input of particles and nutrients due to coastal run-off and the discharge from large rivers. In particular, the Mackenzie River exports huge quantities of sediment into the adjacent Beaufort Sea during summer that are transported further into the Amundsen Gulf and the western part of the Northwest Passage [1]. The Arctic Ocean represents a key area for global carbon [2] and nutrient cycling [3] and is sensitive to climate change. The signs of climate change in the Arctic Ocean include rising temperatures, increasing precipitation, and river discharge coupled with decreasing snow and ice cover [4], [5], [6]. These environmental changes have already had detectable effects on arctic organisms [7], [8].

Viruses are the most abundant biological entities in the oceans [9] and influence global geochemical cycles [10], [11]. Since viral infection is dependent on the abundance of hosts [12], viruses might selectively kill the winners in the competition for nutrients and, thus, are considered to be a driving force in maintaining prokaryotic diversity [13], [14], [15]. As obligate parasites, viruses depend entirely on the host's metabolism for proliferation. Evidence for these tight links between viruses and their hosts have also been found in the Arctic Ocean, where the shrinking ice cover and the increasing levels of sunlight in spring and summer enable substantial primary production [16], and the release of large amounts of organic carbon that in turn stimulate prokaryotic growth and also increase viral abundance [17]–[21]. Viruses are the principal source of prokaryotic mortality in Arctic bottom waters, dwarfing the impact of grazing during winter [22].

Increasingly, flow cytometry (FCM) of fluorescently stained samples is used to determine the abundance of prokaryotes and viruses in aquatic environments [23], [24], [25]. Based on the strength of the fluorescence signal due to staining with nucleic acid dyes, at least two prokaryotic populations can be distinguished by FCM. Since, the intensity of the fluorescence signal has been shown to be proportional to prokaryotic DNA content [26], the populations have been referred to as high (HNA) and low nucleic acid (LNA) cells. Some reports indicate that HNA cells are metabolically most active [27], [28], [29], although LNA cells can be metabolically active too [30]–[33]. Previous studies have found no consistent taxonomic differences between mixed populations of HNA and LNA cells [31], [34]. However, Wang et al. [35] isolated three members of the LNA population that retained their low fluorescence signature in culture, consistent with observations by Vila-Costa et al. [36] who found that the majority of taxa in cytometrically-sorted fractions of HNA and LNA cells were only found in one of the fractions suggesting that HNA and LNA populations are largely composed of different taxonomic groups. Contrary to prokaryotes, the intensity of the fluorescence signal of viruses in FCM analysis does not correlate with genome size [37], although FCM can resolve different populations of viruses. Most marine viruses have relatively low fluorescence intensity and are considered to infect mostly prokaryotes. Viruses with stronger fluorescence signals are thought to primarily infect eukaryotic phytoplankton and other protists [38], [39].

Because of the central role of the Arctic Ocean in global geochemical cycles and its vulnerability to the effects of climate change, understanding the possible consequences of changes in the environment for microbes is critical. Kirchman et al. [40] suggested that, based on a comparison between data from polar regions and low-latitude waters, prokaryotic abundance and growth in the Arctic Ocean appear to be controlled by the supply of dissolved organic matter (DOM) and temperature. Also, Payet and Suttle [20] found that prokaryotic and viral abundance in the Arctic Ocean increased with increasing temperature and the concentration of Chlorophyll-*a* (Chl-*a*), a main source of DOM. However, these studies [20], [40] also show that the effects of temperature and DOM supply on prokaryotes are non-linear, and likely more complex than currently appreciated. Conventional statistical methods such as regression or principal components analysis require data to be transformed (e.g., log-transformation) if they are not normally distributed, else non-parametric tests have to be used. Such methods usually resolve trends in the data, but may miss or obscure the finer picture. An alternative or complementary method of analysis, Artificial Neural Networks (ANNs), constitute a data-driven tool for analyzing and modeling complex relationships. An advantage of ANN-based models is that data do not have to fit pre-defined conditions (e.g., linearity, normal distribution); instead available data are used in a training phase to develop empirical models [41], [42]. For example, ANNs have been used to model phytoplankton [43], [44] and viral production [45], and to predict zooplankton biomass [46].

In this study we used previously published seasonal data from the Arctic Ocean [20] collected from November 2003 to August 2004 to develop ANN-based models of the abundances of prokaryotes and viruses. A number of previous studies have shown that the annual phytoplankton blooms in the Arctic release substantial amounts of DOM that stimulates the growth of heterotrophic prokaryotes [17], [18], [19], [21], consistent with evidence that prokaryotic abundance and growth are limited by the availability of DOM in the Arctic Ocean [40]. Given that phytoplankton are a major source of DOM in arctic waters, we used Chl-*a* as a surrogate parameter for the availability of DOM. The other parameters considered for modeling (temperature, salinity, depth, day length) are easily obtained and representative of changes in the physico-chemical environment of the water column. Thus, potential input parameters for ANN-based models were geared towards capturing potential bottom-up effects on prokaryotic abundance. Although viruses are obligate parasites that depend entirely on the hosts' metabolism for proliferation, we used the same input parameters as for prokaryotes. As most viruses in the ocean infect prokaryotes, using prokaryotic abundance as an additional input parameter might have resulted in better performing ANN-based models of viral abundance. However, we were more interested if changes in the physico-chemical environment and Chl-*a* would predict viral abundance. Also, high-fluorescent viruses are thought to infect phytoplankton [38], [39] so that a direct link between the abundance of this viral group and its potential hosts is represented in the data. The objectives of this study were (1) to identify the most successful combination of parameters (salinity, temperature, depth, day length, Chl-*a*) that lead to the best performing ANN-based models of the abundances of prokaryotes and viruses, and (2) to use these models to further investigate the effects of changes in the environment on prokaryotic and viral abundances by performing simulations using the developed ANN-based models.

## Materials and Methods

### Study area, sampling, and measured parameters

**Figure 1:**
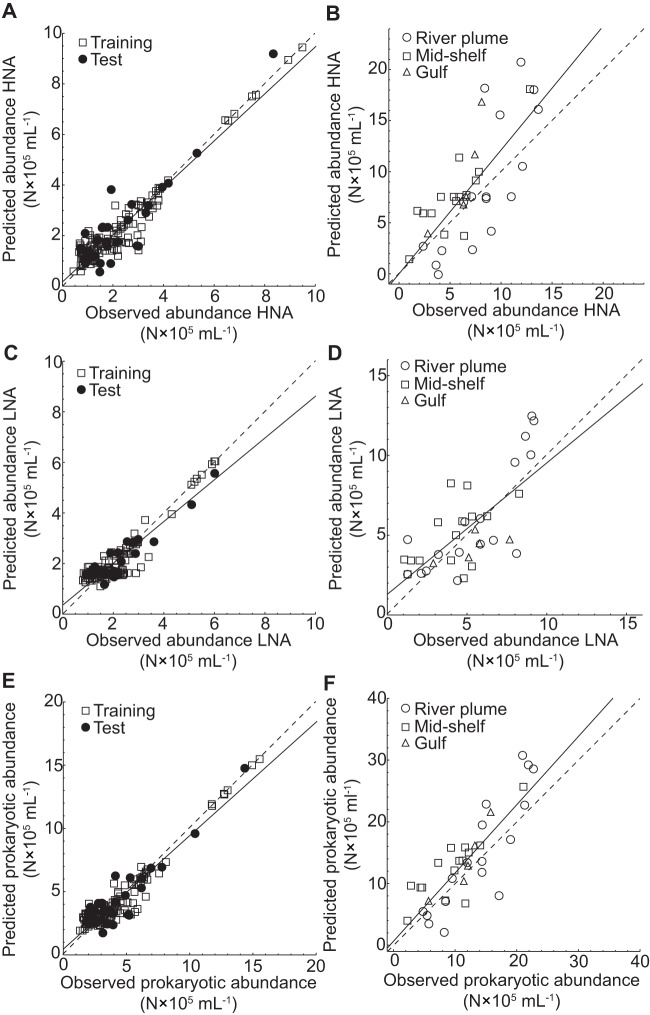
Linear least-squares regression analyses of observed versus predicted prokaryotic abundance. The figure shows the results of the linear least-squares regression analysis computed for the training and test data sets for the abundances of (A) HNA, (C) LNA, and (E) total prokaryotic abundance (*r* ^2^ = 0.898; *y* = 0.456+0.896 *x*). Additionally, the results of the spatial data set (region designations as in [20]) used for evaluating the trained ANNs are shown for the abundances of (B) HNA, (D) LNA, and (F) total prokaryotic abundance (*r* ^2^ = 0.703; *y* = 0.621+1.107 *x*). Solid lines represent the linear least-squares fit to the data and dashed lines the theoretical 1∶1 fit. The parameters for the linear least-squares regression analyses for panels A–D can be found in Table 3.

The data used for modeling are from Payet and Suttle [20], and were collected from 8 stations in the south-eastern Beaufort Sea of the Canadian Arctic. From 4 November 2003 to 6 August 2004 seasonal samples were retrieved 21-times at roughly weekly intervals from depths of 3 m, 10 m, 20 m, 30 m, 50 m, 100 m, 150 m, and 220 m at a station in Franklin Bay (70°03′ N, 126°30′ W); occasional sampling problems led to 156 samples being recovered. Additionally, 37 samples collected between the surface and a maximum depth of 80 m (bottom depth permitting) at 7 stations between the Mackenzie River and Amundsen Gulf (between 69.5°–71.5° N and 122.3°–138.6° W) from 4 July to 10 August 2004 (see Fig. 1 in Payet and Suttle [20]) provided a spatial data set. In addition to physico-chemical parameters such as temperature and salinity, data were collected on Chl-*a* and prokaryotic and viral abundances. FCM was used to distinguish HNA and LNA prokaryotic cells, and high- (V1) and low-fluorescent (V2) viruses, based on the fluorescence intensity after staining with the nucleic acid dye SYBR Green I. Auto- and heterotrophic prokaryotic cells were not distinguished from each other so that total prokaryotic abundance (the sum of HNA and LNA cells) includes all prokaryotic cells. More details on the sampling scheme and the measured parameters are given by Payet and Suttle [20].

### Modeling prokaryotic and viral abundances using ANNs

#### Data preparation

Eighty percent of the seasonal data were used for training the networks and the remainder (test data) were used exclusively to determine when the training had finished (see below). The spatial data set, comprised of 37 samples, was used to evaluate the trained networks in order to determine the best performing ANN-based model. The following 5 input parameters were considered: Chl-*a* (µg L^−1^), day length (hours), depth (m), salinity (psu), and temperature (°C). The day length, defined as the time in hours from sunrise to sunset, was calculated based on the sampling date and the coordinates of the stations using the software XEphem (version 3.7.4, Clear Sky Institute). Prior to training, all data were scaled to a mean of zero and unity variance.

#### Modeling strategy

A short introduction to ANNs is provided as part of the online supporting information (Text S1, Fig. S1). For an in depth introduction to ANNs we refer to Basheer and Hajmeer [42]. The input parameters were used alone and in combination with up to two other parameters to develop ANN-based models of the abundances of HNA and LNA cells (10^5^ mL^−1^), and of V1 and V2 viruses (10^6^ mL^−1^). Feed-forward (FFW) ANNs and radial basis function (RBF) ANNs with one layer of hidden neurons and one output neuron were implemented in Mathematica (version 7.0.1) using the Neural Networks application package (version 1.1; both from Wolfram Research). Bias terms with a fixed value of 1 were included in the input and the hidden layer for FFW networks and in the output layer for RBF networks. Before training, the parameters of the networks were initialized using the option “LinearParameters” to randomize the initial values of the non-linear parameters within the range of the input data and to completely randomize the linear parameters. We used the Levenberg-Marquardt algorithm [41], [47] to train ANNs with 2–15 hidden neurons each for 100 iterations, employing the sigmoid function as the activation function of the hidden units for FFW networks and the exponential function for RBF networks. The progress of training was monitored using the root-mean-squared error (RMSE) of the networks.

The initial values of the weights of the network can influence the outcome of the training procedure [41]. Thus, in the first phase of training, for each set of in- and output parameters and number of hidden units, 100 ANNs were initialized and trained as described above. ANNs can memorize the training data if trained for too many iterations (over-training), particularly when the networks have a large number of hidden units. Over-training makes predictions based on new input data inaccurate and can be prevented by cross-validation [41]. As described above, the data were split into training and test data sets. While the training data were used for adjusting the networks parameters in order to decrease the networks error in the subsequent iteration, the test data were only used to validate the network at each iteration during training without interfering in parameter adjustment. Training was assumed to have converged when the sum of the RMSE of the training and test data sets reached a minimum. The best performing ANNs for each set of in- and output parameters, number of hidden neurons, and ANN type were determined by searching for the smallest sum of the RMSE of the training and validation data sets at convergence of training. In order to further explore the parameter space for the best possible solutions, the best performing network architectures from the first phase were initialized, trained, and screened another thousand times as described above. The best-performing networks from this second phase of training were reconstituted at the iteration of the minimum of the combined RMSE by retrieving the corresponding network parameters from the training record.

#### Model evaluation

The ANN-based models from the second phase of training were evaluated using the spatial data by performing linear least-squares regression analysis between observed and predicted abundances of HNA, LNA, V1, and V2. The best performing network architecture for each output parameter was chosen based on the parameters of the linear least-squares regression analysis calculated between observed and predicted values of the spatial data set, i.e., the slope (*k*) closest to 1 given a co-efficient of determination *r* ^2^>0.5. Total prokaryotic and viral abundances were obtained by summing the model outputs for the abundances of HNA and LNA cells or V1 and V2 viruses, respectively.

#### Simulation of the abundances of prokaryotes and viruses

The ANN-based models were used to simulate the abundances of prokaryotic and viral populations as well as total prokaryotic and viral abundances. To obtain the simulations, the values of the input parameters were varied within the range of the seasonal data; specifically, temperature varied between −1.8–2.8°C, day length between 0–24 hours, and Chl-*a* from 0.01–0.61 µg L^−1^. The abundances of V1 and V2 viruses as well as total viral abundance was simulated at 5 m, 50 m, 100 m, 150 m, and 200 m depth.

### Statistical analyses

Statistical analyses were performed with Mathematica 7.0.1 (Wolfram Research). Linear least-squares regression was used to determine the relationships between observed versus predicted abundances of HNA, LNA, V1, and V2. Differences in the *y*-offset and the slope of linear least-squares regression analysis against the theoretical values of 0 and 1, respectively, were tested by calculating the *t*-statistic according to the equation *t* = |(*b_yx_*–*B_yx_*)|/|(*Sb_yx_*–*SB_yx_*)|, where *b_yx_* and *B_yx_* represent the *y*-offsets or slopes and *Sb_yx_* and *SB_yx_* the respective standard deviations. Due to non-normality of the data, seasonal and spatial data were log-transformed before statistical analyses except for temperature, salinity, and day length, which were not transformed. A Student's *t*-test was used to test for differences in the parameters between the seasonal and spatial data sets. Stepwise multiple linear regression (SMLR) was performed to obtain statistical models of the abundances of HNA and LNA cells as well as V1 and V2 viruses as a comparison to the ANN models. Similarly to the ANN-based models, the SMLR models were developed using the seasonal data whereas the spatial data were used for evaluation. The *p*-values are reported for the two-tailed *t*-distribution. Results of statistical tests were assumed to be significant at *p*-values ≤0.05.

## Results

### Differences between seasonal and spatial data sets

Detailed statistical analyses on temporal, depth, and spatial variations in the data can be found in Payet and Suttle [20]. Here, we focus on differences between the seasonal and spatial data that are relevant for the modeling approach. Differences between the parameters in the seasonal and spatial data (Tables 1, 2) were statistically significant for every parameter (Student's *t*-test: in every case *p*<0.0004). The spatial data were gathered during the arctic summer under almost continuous sun light, higher water temperatures, and from shallower waters than for the seasonal data. On average, salinity was lower in the spatial data set compared to the seasonal data set, indicating a stronger freshwater influence. All biological parameters (Chl-*a*, abundances of prokaryotes and viruses) were on average lower in the seasonal data set than in the spatial data set (Tables 1, 2). In summary, the spatial data set for evaluating the ANN-based models differed from the data used to train the networks and, thus, provides a good test for the models' performance.

**Table 1:** Parameters measured as part of the seasonal data set.

Parameter	*Avg*	*SD*	Minimum	Maximum	*CV*	*N*
Depth	74.8	74.7	1	225	99.9	156
Temperature	−1.20	0.69	−1.73	2.78	57.2	156
Salinity	31.90	1.63	27.31	34.69	5.1	156
Day length	12.6	8.4	0.0	24.0	66.7	156
Chl-*a*	0.08	0.10	0.01	0.61	132.7	156
HNA cells	2.22	1.62	0.44	9.49	72.9	156
LNA cells	2.05	1.08	0.80	6.04	53.0	156
Prokaryotes	4.27	2.68	1.32	15.54	62.7	156
V1 viruses	0.93	0.81	0.16	4.08	87.4	156
V2 viruses	5.26	3.17	0.90	16.11	60.4	156
Viruses	6.18	3.92	1.27	19.96	63.4	156

The average (*Avg*), standard deviation (*SD*), minimum, maximum, coefficient of variation (*CV*; %), and number of samples (*N*) are given. Depth (m), temperature (°C), salinity, day length (hours), Chl-*a* (µg L^−1^), the abundance of HNA and LNA cells as well as total prokaryotic abundance (N×10^5^ mL^−1^), and the abundance of V1 and V2 viruses as well as total viral abundance (N×10^6^ mL^−1^).

**Table 2:** Parameters measured as part of the spatial data set.

Parameter	*Avg*	*SD*	Minimum	Maximum	*CV*	*N*
Depth	24.5	21.7	1	78	88.1	37
Temperature	1.04	2.93	−1.45	8.52	282.5	37
Salinity	28.78	4.33	15.84	32.67	115.0	37
Day length	23.3	1.7	19.2	24.0	7.2	37
Chl-*a*	0.49	0.60	0.07	2.37	121.6	37
HNA cells	6.84	3.31	1.01	13.65	48.4	37
LNA cells	4.93	2.44	1.06	9.19	49.5	37
Prokaryotes	11.77	5.59	2.28	22.71	47.5	37
V1 viruses	1.86	1.44	0.06	5.35	77.4	37
V2 viruses	12.80	5.75	2.08	23.20	44.9	37
Viruses	14.68	6.94	2.49	28.55	47.3	37

The average (*Avg*), standard deviation (*SD*), minimum, maximum, coefficient of variation (*CV*; %), and number of samples (*N*) are given. Depth (m), temperature (°C), salinity, day length (hours), Chl-*a* (µg L^−1^), the abundance of HNA and LNA cells as well as total prokaryotic abundance (N×10^5^ mL^−1^), and the abundance of V1 and V2 viruses as well as total viral abundance (N×10^6^ mL^−1^).

### ANN-based models of the abundances of prokaryotic and viral populations

#### Model development

ANN-based models of the abundances of HNA and LNA cells as well as V1 and V2 viruses were developed using 7 different combinations of the input parameters for FFW and RBF networks (Tables S1, S2, S3, S4, S5, S6, S7, S8). The results of the first two phases of model development consistently indicated that these 7 combinations were best performing for all ANN-based models. Based on the combined RMSE of the training and test data set, there was considerable variation in the performance of the various ANN-based models. The RMSE of the models of HNA cells varied between 0.54 and 0.82 and between 0.63 and 0.94 for FFW and RBF networks, respectively (Tables S1 and S3). For models of the abundance of LNA cells, the RMSE ranged from 0.61 to 0.86 and from 0.75 to 1.05 for FFW and RBF networks, respectively (Tables S2 and S4). The RMSE for FFW and RBF networks modeling the abundance of V1 cells varied between 0.45 and 0.92 and between 0.52 and 0.91, respectively (Tables S5 and S7). For FFW and RBF models of the abundance of V2 viruses, the RMSE varied between 0.55 and 0.86 and between 0.60 and 0.92, respectively (Tables S6 and S8).

#### Best performing ANN-based models

The ANN-based models developed in this study are available from the authors in the form of Mathematica source files. The best performing network model of the abundance of HNA cells as evaluated using the spatial data used Chl-*a* and temperature as the input parameters to a FFW network with 14 hidden units (Table 3, Fig. 1A–B). The model explained 90% of the variation of HNA cells in the seasonal and 56% of the variation in the spatial data set (Table 3). The abundance of LNA cells was modeled best by a RBF network with 15 basis functions and Chl-*a* and temperature as input parameters (Table 3, Fig. 1C–D), which explained 84% and 52% of the variation in the seasonal and spatial data sets, respectively (Table 3). The sum of the abundances of HNA and LNA cells obtained from the ANN-based models was similar to observed prokaryotic abundances for the seasonal (Fig. 1E; *r* ^2^ = 0.898) and spatial data sets (Fig. 1F; *r* ^2^ = 0.703).

**Table 3:** Best performing ANN-based models.

Input parameters	Output parameter	Network	Hidden units or basis functions	RMSE	*r* ^2^	*r* ^2^-spatial	Intercept	Intercept spatial	*k*	*k*-spatial
Chl-*a*, temperature	HNA	FFW	14	0.706	0.901	0.559	0.169	−0.001	0.929	1.213
Chl-*a*, temperature	LNA	RBF	15	0.808	0.843	0.516	0.357	1.294	0.826	0.824
Chl-*a*, day length, depth	V1	RBF	15	0.522	0.951	0.586	0.045	0.691	0.959	1.205
Chl-*a*, day length, depth	V2	FFW	8	0.669	0.905	0.544	0.455	3.728	0.915	0.781

**Figure 2:**
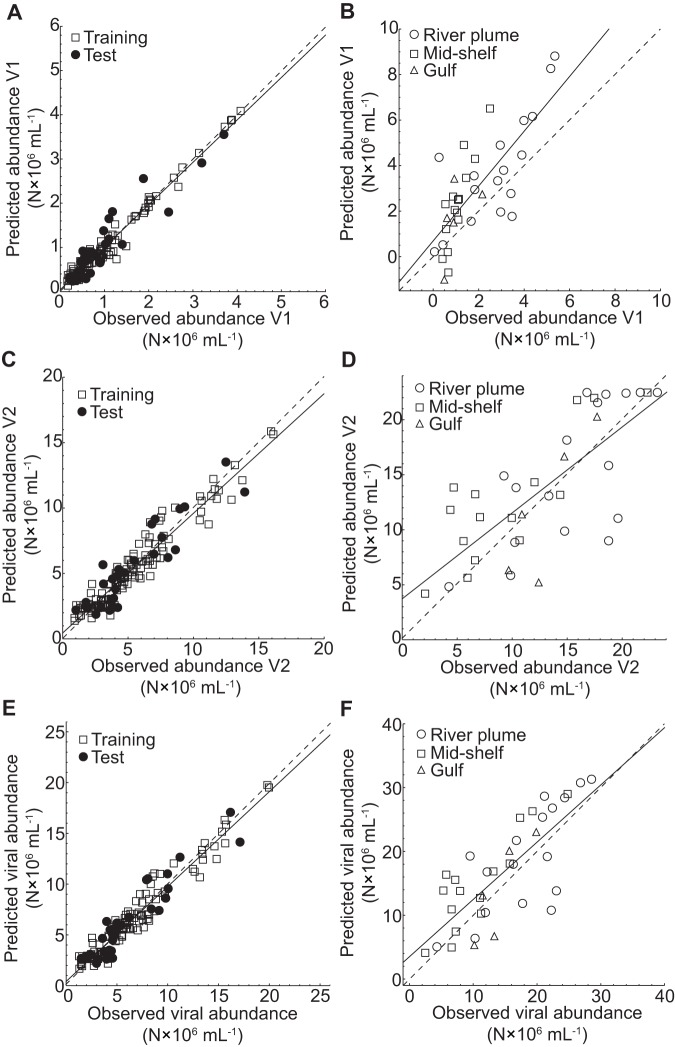
Linear least-squares regression analysis of observed versus predicted viral abundances. The figure shows the results of the linear least-squares regression analysis computed for the training and test data set of the abundances of (A) V1 viruses, (C) V2 viruses, and (E) total viral abundance (*r* ^2^ = 0.929; *y* = 0.425+0.934 *x*). Additionally, the results of the spatial data set (region designations as in [20]) used for evaluating the trained ANNs are shown for the abundances of (B) V1 viruses, (D) V2 viruses, and (F) total viral abundance (*r* ^2^ = 0.599; *y* = 3.495+0.897 *x*). Solid lines represent the linear least-squares fit to the data and dashed lines the theoretical 1∶1 fit. The parameters for the linear least-squares regression analyses can be found in Table 3.

The table gives the in- and output parameters, the network type (Feed-Forward Artificial Neural Network: FFW; Radial Basis Function Artificial Neural Network: RBF), the number of hidden units for FFW and number of radial basis functions for RBF, and the root-mean-squared error of the networks (RMSE) summed up for the training and test data set at convergence of the training procedure for the best performing ANN-based models as evaluated using the evaluation data set. Additionally, the coefficient of determination (*r* ^2^), the *y*-axis intercept, and the slope (*k*) of the linear least-squares regression analysis between observed and predicted values computed for the combined training and test data set as well as for the spatial data set are shown (see also Figs. 1, 2).

The best performing model for the abundance of V1 viruses was a RBF network with 15 basis functions using Chl-*a*, day length, and depth as input parameters (Table 3, Fig. 2A–B). The model of the abundance of V1 viruses explained 95% and 59% of the variation in the seasonal and spatial data sets, respectively (Table 3). The chosen model for the abundance of V2 viruses consisted of a FFW network with 8 hidden units employing the same input parameters as the model for V1 viruses and explained 91% and 54% of the variation in the seasonal and spatial data, respectively (Table 3, Fig. 2C–D). Total viral abundance was calculated by summing the abundances of V1 and V2 viruses obtained from the models. The fit between observed and predicted viral abundances was comparable to the models of the abundances of the viral populations explaining 93% of the variation in the seasonal (Fig. 2E) and 60% in the spatial data set (Fig. 2F).

In summary, although the ANN classes and their structures differed between the models for HNA and LNA cells as well as for V1 and V2 viruses, the models for the prokaryotic and viral populations used the same input parameters (Table 3). Also, in every case (Figs. 1, 2) the slope and the *y*-axis intercept for the linear least-squares regressions between observed and predicted abundances were not significantly different from 1 and 0, respectively (in every case: *p*>0.05).

#### Comparison between ANN and SMLR models

The best performing SMLR models for the abundances of HNA and LNA cells consisted of Chl-*a*, salinity, day length, and temperature (Table 4). However, despite the higher number of parameters in the SMLR models for the abundances of the two prokaryotic populations, the performance of these models based on *r* ^2^-values was inferior to the ANN models, for both the seasonal and spatial data sets (Tables 3, 4). The abundance of V1 viruses was best modeled by a SMLR model consisting of Chl-*a*, day length, and salinity instead of depth as compared to the ANN-based model. The best performing SMLR and ANN-based models of the abundance of V2 viruses used the same input parameters; however, the ANN models of the abundances of V1 and V2 viruses were superior to the SMLR models (Tables 3, 4).

**Table 4:** Stepwise multiple linear regression (SMLR) analysis of the abundances of HNA and LNA cells as well as of V1 and V2 viruses.

Parameters	*F*-ratio	*r* ^2^	*p*	*r^2^*-spatial	Coefficient	*t*-statistic	*SE*	*p*
HNA	380.4	0.71	<0.0001	0.36				<0.0001
*y*-intercept					2.976	10.4	0.286	<0.0001
Chl-*a*					0.283	9.8	0.029	<0.0001
Salinity					−0.072	−7.7	0.009	<0.0001
Day length					0.007	5.0	0.001	<0.0001
Temperature					0.089	4.7	0.019	<0.0001
LNA	245.5	0.61	<0.0001	0.35				<0.0001
*y*-intercept					1.722	7.2	0.239	<0.0001
Chl-*a*					0.197	8.2	0.024	<0.0001
Day length					0.007	6.1	0.001	<0.0001
Temperature					0.078	5.0	0.016	<0.0001
Salinity					−0.037	−4.7	0.008	<0.0001
V1	522	0.77	<0.0001	0.37				<0.0001
*y*-intercept					2.970	11.0		<0.0001
Chl-*a*					0.346	11.2		<0.0001
Salinity					−0.087	−9.4		<0.0001
Day length					0.009	5.8		<0.0001
V2	362.1	0.70	<0.0001	0.27				<0.0001
*y*-intercept					1.128	26.2	0.043	<0.0001
Depth					−0.233	−9.0	0.026	<0.0001
Day length					0.009	6.5	0.001	<0.0001
Chl-*a*					0.175	5.4	0.033	<0.0001

The table gives the parameters for the best performing SMLR models and their coefficients. Additionally, the coefficient of determination using the SMLR model developed with the seasonal data set (*r* ^2^) as evaluated using the spatial data set (*r* ^2^-spatial) is given.

### Simulating the abundances of prokaryotes and viruses

**Figure 3:**
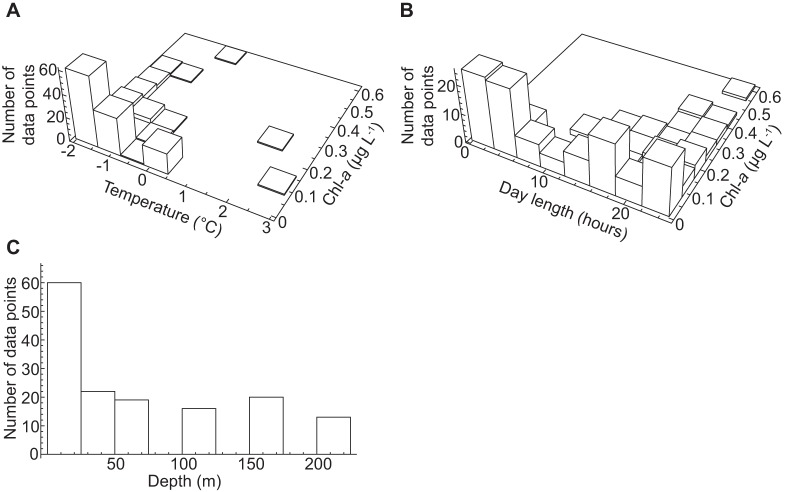
Data frequency distribution. The figure shows the frequency distribution of the seasonal data (comprised of the training and test data) over the parameter space used in the ANN-based simulations. The distributions for (A) temperature and Chl-*a*, (B) day length and Chl-*a*, and (C) depth are shown.

To facilitate the interpretation of the simulations of the abundances of HNA and LNA cells as well as of V1 and V2 viruses using the ANN-based models it is important to consider the distribution of data over the parameter space (Fig. 3). The ANN-based models were well supported by data below temperatures of 0.5°C and up to 0.5 µg L^−1^ of Chl-*a*, with the highest number of data points at lowest temperatures and values of Chl-*a* (Fig. 3A). The entire range of day lengths was well represented (Fig. 3B). Most data were available from the surface to 25 m depth with a lack of data around 85 m, 140 m, and 185 m (Fig. 3C).

**Figure 4:**
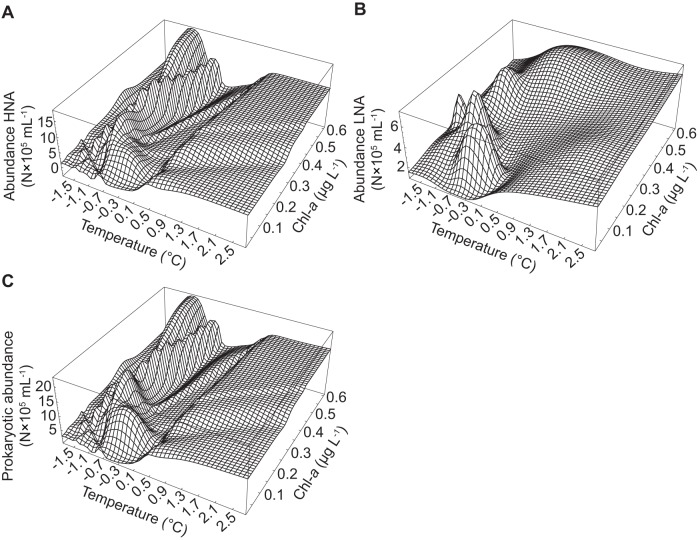
Simulation of prokaryotic abundance. The figure shows the abundances of (A) HNA, (B) LNA, and (C) total prokaryotic abundance. The ANNs described in Table 3 were used to simulate the abundances of HNA and LNA cells at temperatures ranging from −1.8–2.8°C and Chl-*a* ranging from 0.01–0.61 µg L^−1^. Total prokaryotic abundance was computed by summing the simulated abundances of HNA and LNA cells.

Overall, the abundances of HNA cells and total prokaryotes exhibited a maximum at temperatures ranging between −1.3 and −0.3°C with comparatively small effects of changes in Chl-*a* (Fig. 4A and C). Between 0.5 and 2.8°C, the abundances of HNA cells and total prokaryotes increased slightly with increasing Chl-*a*. Overall, the abundance of LNA cells increased with increasing temperature and Chl-*a* and showed 4 pronounced peaks at temperatures below 0°C.

**Figure 5:**
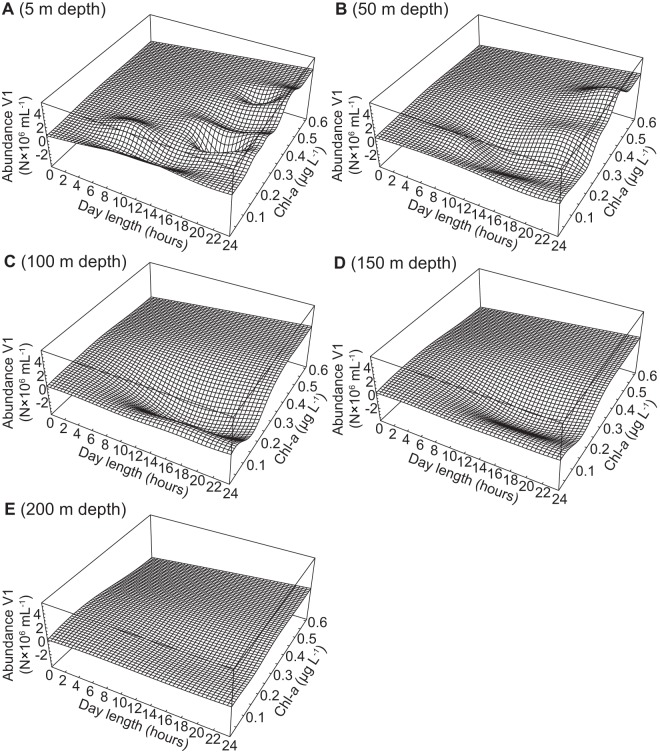
Simulation of the abundance of V1 viruses. The figure shows the abundance of V1 viruses at a depth of (A) 5 m, (B) 50 m, (C) 100 m, (D) 150 m, and (E) 200 m. The ANN described in Table 3 was used to simulate the abundance of V1 viruses at day lengths ranging from 0–24 hours and Chl-*a* from 0.01–0.61 µg L^−1^.

**Figure 6:**
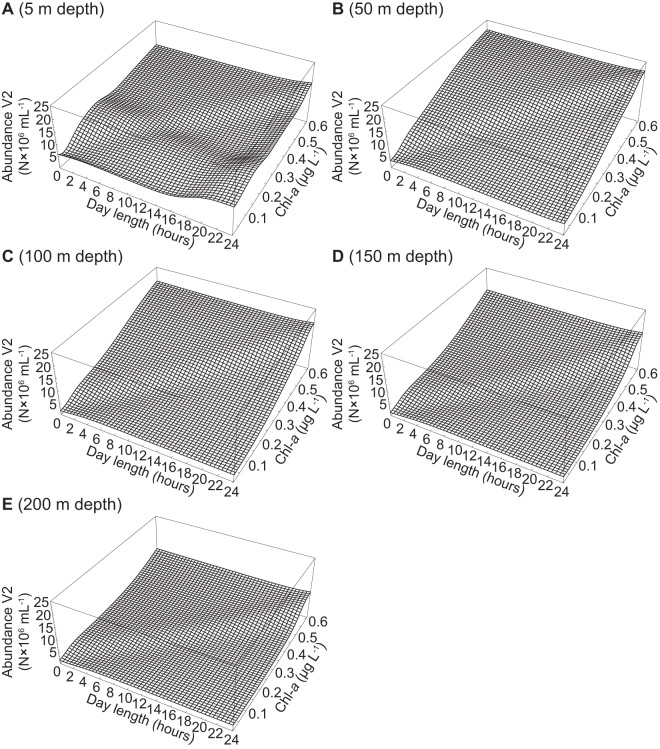
Simulation of the abundance of V2 viruses. The figure shows the abundance of V2 viruses at a depth of (A) 5 m, (B) 50 m, (C) 100 m, (D) 150 m, and (E) 200 m. The ANN described in Table 3 was used to simulate the abundance of V2 viruses at day lengths ranging from 0–24 hours and Chl-*a* from 0.01–0.61 µg L^−1^.

**Figure 7:**
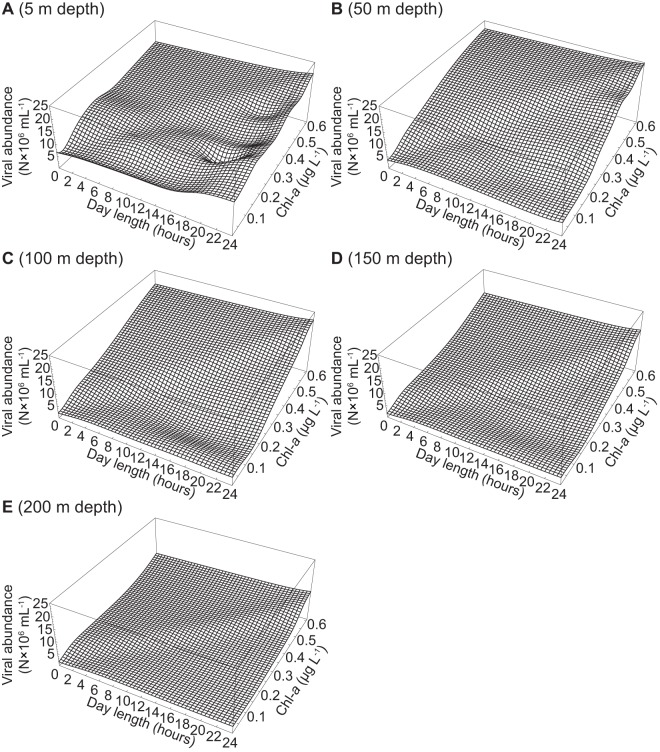
Simulation of viral abundance. The figure shows total viral abundance at a depth of (A) 5 m, (B) 50 m, (C) 100 m, (D) 150 m, and (E) 200 m. The ANNs described in Table 3 were used to simulate the abundances of V1 and V2 viruses at day lengths ranging from 0–24 hours and Chl-*a* from 0.01–0.61 µg L^−1^. Total viral abundance was computed by summing the simulated abundances of V1 and V2 viruses.

The abundance of V1 viruses increased slightly with increasing day length and Chl-*a* at 5 m and 50 m depths (Fig. 5A–B). At 100 m and 150 m depths, the abundance of V1 viruses was predicted to be negative at Chl-*a* concentrations of 0.1 and 0.3 µg L^−1^ and day lengths between 8–24 hours (Fig. 5C–D). The abundance of V1 viruses decreased with increasing Chl-*a* at 200 m depth and was negative, particularly at short day lengths (Fig. 5E). Highest abundances of V1 viruses were reached in surface waters at 24 hour day length and between 0.4 and 0.5 µg L^−1^ Chl-*a* (Fig. 5A–B). The abundances of V2 viruses and of total viruses were mostly influenced by Chl-*a* and depth (Figs. 6, 7). Overall, V2 viruses and total viral abundance increased with increasing Chl-*a*, and were highest at Chl-*a* values around 0.6 µg L^−1^, 24 hour day length, and 50 m depth (Figs. 6, 7). The effects of Chl-*a* on the abundances of V2 and total viruses were mitigated by increasing depth (Figs. 6, 7).

## Discussion

### Ecological interpretation of the ANN-based models of prokaryotes and viruses

#### Availability of data

As a data-driven modeling approach, ANNs rely on the availability of suitable data. For the Arctic Ocean, seasonal data on prokaryotic and viral abundances over depth are very scarce. For a meaningful interpretation of the simulations performed with the ANN-based models (Figs. 4, 5, 6, 7), it is important to note that the data was not equally distributed over the entire range used in the simulations (Fig. 3). Thus, in regions where no data were available for training, the ANNs were unable to use an error measurement for improvement during training, so the predictions are based on extrapolations. Although we took great care to develop ANN-based models with a high ability to generalize, regions in the parameter space used for the simulations with an apparent lack of data will not be taken into account for further interpretation.

#### Differences among the models for prokaryotic and viral populations

The choice of temperature and Chl-*a* for modelling the abundances of HNA and LNA cells (Fig. 1) confirms the conclusions of other authors [20], [40], that the abundance and growth of heterotrophic prokaryotes in the Arctic Ocean is limited by temperature and the availability of DOM, since phytoplankton is a major source of DOM in the Arctic Ocean. However, if the reactions to changes in temperature and Chl-*a* of HNA and LNA cells are governed by the same mechanisms, the results of the simulations (Fig. 4A–B) should be similar and the same class of ANNs should have been able to perform equally well for both populations. However, the results show that modeling the abundance of LNA cells using the same input parameters with an FFW network (*r* ^2^-spatial; Table S2) would result in a model that performs much worse than one employing an RBF network (Table 3). Therefore, although the abundances of HNA and LNA cells appear to be limited by temperature and the availability of DOM, the mechanisms governing the response are clearly different for the two populations (Fig. 4A–B).

Although the literature is not conclusive with respect to the biological basis of the distinction between HNA and LNA cells [27], [30], [33], at least two not mutually exclusive mechanisms might be responsible for the differences in our models. Assuming that more abundant prokaryotes such as HNA cells are better competitors for nutrients, the differences in the models might be explained by competition between HNA and LNA cells. Also, given that flagellate grazing appears to be negligible in the Arctic Ocean [22], the primary source of prokaryotic mortality is viral lysis. Viral infection is dependent on the abundance [12] and/or activity [48] of suitable hosts. Thus, differences in growth rates and abundances between the two prokaryotic groups might lead to differences in viral lysis and differences among our models.

Similar to the models for prokaryotes, the reaction to changes in depth, day length and Chl-*a* as the optimal set of input parameters differed among the models for V1 and V2 viruses (Figs. 5, 6). Additionally, a FFW network using the same input parameters modeling the abundance of V1 viruses would not have performed well when evaluated with the spatial data set (*r* ^2^-spatial; Table S5). Likewise, using a RBF network to model the abundance of V2 viruses also resulted in unsatisfactory results with the evaluation data set (*r* ^2^-spatial; Table S8). V1 and V2 viruses are distinct groups of viruses with V1 viruses thought to primarily infect eukaryotic phytoplankton [38], [39]. Thus, it makes sense that Chl-*a* was an input parameter for the model of V1 viral abundance, as it should link V1 viruses to their hosts. More surprising was that V2 viruses, which are assumed to infect mostly prokaryotes, would have the same input parameters. Nevertheless, changes in depth, day length, and Chl-*a* will only indirectly affect the abundance of V2 viruses, explaining the differences between the models for V1 and V2 viruses. This is consistent with reports that different marine viral groups distinguished by FCM respond differently to environmental changes [49].

#### Ecological significance of input parameters

The simulations using the ANN-based models developed in this study suggest that small changes in temperature explained most of the variation, especially for the abundance of HNA cells (Fig. 4). Chl-*a*, as a surrogate parameter for DOM supply, affected the abundances of HNA and total prokaryotes, mostly at temperatures between −1.3 to −0.3°C (Fig. 4A and C). Also, the effect of temperature on the abundances of HNA cells and total prokaryotes were not only positive as suggested by correlation analysis [20]. Rather, the abundances of prokaryotes and HNA cells initially increased with temperature and then decreased again at temperatures of about −0.7 to −0.3°C. Thus, the model captured the time-lag between the phytoplankton bloom and the increase in prokaryotic abundance in surface waters and also the effect of the subsurface chlorophyll maximum during summer where temperatures were lower year round [20]. Phytoplankton constitutes a major source of DOM in the Arctic Ocean [21]; however, the input of DOM due to large rivers [1] in the warmer season cannot be neglected. Thus, the lack of a positive relationship between Chl-*a* and prokaryotic abundance at temperatures above −0.3°C might indicate that under these conditions most of the DOM is due to import from terrestrial sources. Nevertheless, the models are not well constrained at temperatures above −0.3°C (Fig. 3A) so that the model predictions above this temperature need to be considered cautiously.

In the Arctic Ocean, the abundance of viruses increases with increasing prokaryotic abundance as a consequence of phytoplankton production [17], [20], [21]. Thus, it is not surprising that Chl-*a* was picked as one of the input parameters for models of viral abundance (Figs. 5, 6, 7). Additionally, viral abundance generally declines with depth, an effect that was also captured by our models. However, day length was an unexpected choice as one of the input parameters for the models of viral abundance, although most of the variability in the abundance of viruses was explained by Chl-*a* and depth with the exception of surface waters. Metabolically highly active host organisms sustain high viral abundances and viral abundance generally scales with the productivity of the system [15], [50]; hence, the choice of day length is likely because phytoplankton production depends on light. The effect of changes in day length and Chl-*a* on the abundance of V1 viruses appears to be direct since phytoplankton are the probable hosts for this group of viruses. However, the effects of changes in day length and Chl-*a* on the abundance of V2 viruses, mostly infecting prokaryotes, is likely an indirect effect via the release of DOM from phytoplankton.

### Critical evaluation of the modeling approach

The biggest strength of ANNs is their high parallelism, that is the number of hidden units for FFW networks or number of basis functions in the case of RBF networks, making ANNs relatively immune to outliers in the data. However, this strength can be the biggest problem if the objective is to seek models with a high ability to generalize based on new data [41]. Thus, ANNs can be made to fit any kind of arbitrary data simply by increasing the number of processing units (hidden units, radial basis functions) and training iterations. However, the result would essentially be a data lookup table with very little to no predictive value. Thus, we employed cross-validation to avoid over-training and over-parameterization of the ANNs. The rationale behind this approach is that an additional test data set is used during model development. At the onset of training, the error of the network will decline with every training iteration, as it does for the training data set that is used to adjust the network's parameters. However, when the ANN starts to memorize the training data, the error for the test data set will increase, indicating that training should stop. Likewise, over-parameterized ANNs can be distinguished by comparing the errors between different ANNs that contain different numbers of processing units. Initially the error of the trained ANNs will decline with increasing numbers of processing units but eventually will increase again, especially for the test data, thus, allowing to constrain the ANNs to the optimal number of processing units. Although this strategy should result in well trained ANN-based models with high generalization ability we used an additional evaluation data set, comprised of the spatial data (Table 2), to further evaluate the trained ANNs. The spatial data used to evaluate the trained ANNs was on average significantly different from the training data. Thus, the evaluation data further ensured that only well-trained ANNs with a high ability to generalize were selected, as indicated by significant (*p*≤0.05) and relevant (−0.5>*r* ^2^>0.5) *r* ^2^-values for the linear least-squares regression analyses between observed and predicted values for the seasonal and spatial data sets calculated for all ANN-based models (Table 3). Also, the fit between observed and predicted values for the seasonal and spatial data for all ANN-based models was statistically indistinguishable from one-to-one, indicating successful model development (Figs. 1, 2). Nevertheless, the scatter of the data around the regression lines was higher for the spatial data set as indicated by the lower *r* ^2^-values compared to the seasonal data set (Figs. 1, 2, Table 3). This indicates that although the ANN-based models' performance when confronted with new data is good, and better than the SMLR models (Tables 3, 4), the ability to generalize comes at a performance cost. This is particularly evident when the ANN-based models predicted negative abundances for prokaryotes and viruses at specific values of the input parameters (Fig. 1B, Fig. 2B, Fig. 5). The ANNs were not forced to predict only positive values, and the linear least squares regressions were not forced through the origin (Figs. 1, 2); thus, at very low abundances and/or combinations of input parameters that were not found in the environment, the ANN-based models sometimes predicted negative abundances.

In principle, the developed ANN-based models might also be useful to predict changes in prokaryotic and viral abundances as the Arctic Ocean warms. However, model outputs depended on two (HNA, LNA) and three (V1, V2) input parameters and ANNs for viral abundances were independent of temperature as an input parameter (Table 3). Since warmer waters in the Arctic Ocean will likely also have an effect on Chl-*a* and the models are not well constrained for high temperatures (Fig. 3A), such predictions need to be interpreted cautiously.

### Summary and conclusions

The data in Payet and Suttle [20] detailed seasonal and spatial changes in the abundances of prokaryotes and viruses in the Arctic Ocean in the context of environmental data. Based on these data we demonstrated that it is possible to model the temporal development of the abundances of prokaryotes and viruses in the Arctic Ocean using ANNs and that these models are superior to SMLR models. The abundances of HNA and LNA cells were best modeled using temperature and Chl-*a* as input parameters, while the best models of V1 and V2 viral abundances used depth, Chl-*a*, and day length as input parameters. The models between the groups of prokaryotes and viruses differed in the ANN classes used (FFW versus RBF networks, respectively) and responded differently to changes in the input parameters. Together, these results indicate that the mechanisms governing the reaction to changes in the environment as represented by the respective input parameters differed among prokaryotic and viral populations. Thus, the FCM-based distinction between HNA and LNA cells as well as V1 and V2 viruses appears to be ecologically relevant.

The general trends of decreasing viral abundance with increasing depth and decreasing productivity of the system were captured well by the ANN-based models. Since phytoplankton production depends on light, the combination of Chl-*a* and day length appears to represent changes in productivity in the virus abundance models. In the Arctic Ocean, because phytoplankton are a major source of DOM [21], Chl-*a* can be interpreted as a proxy for DOM supply. The models show that temperature was the main factor explaining most of the variation in the abundance of HNA cells and total prokaryotic abundance.

## Supporting Information

Figure S1 **Schematic description of Artificial Neural Networks** (**ANNs**)**.** The figure details the network architectures of feed-forward (FFW) and radial basis function (RBF) ANNs used in this study. Data is fed into the input units (x_1_...x_n_) and transmitted along the weights to the hidden layer. The activation function for hidden units of FFW ANNs was the sigmoid function (σ) and for RBF ANNs the gaussian function was used. The output of the ANNs (ŷ) is compared to the known target values (y) and the difference is computed as the root-mean-squared error (RMSE). Bias terms are omitted for simplicity.
(PDF)

Table S1 **Feed-forward artificial neural network** (**FFW**)**-based models of the abundance of HNA cells.** The table gives the input parameters, the number of hidden units, and the root-mean-squared error of the networks (RMSE) summed up for the training and test data set at convergence of the training procedure. Additionally, the coefficient of determination (*r^2^*), the *y*-axis intercept, and the slope (*k*) of the linear least-squares regression analysis between observed and predicted values computed for the combined training and test data set as well as for the spatial data set are shown.
(PDF)

Table S2 **Feed-forward artificial neural network** (**FFW**)**-based models of the abundance of LNA cells.** The table gives the input parameters, the number of hidden units, and the root-mean-squared error of the networks (RMSE) summed up for the training and test data set at convergence of the training procedure. Additionally, the coefficient of determination (*r^2^*), the *y*-axis intercept, and the slope (*k*) of the linear least-squares regression analysis between observed and predicted values computed for the combined training and test data set as well as for the spatial data set are shown.
(PDF)

Table S3 **Radial basis function artificial neural network** (**RBF**)**-based models of the abundance of HNA cells.** The table gives the input parameters, the number of basis functions, and the root-mean-squared error of the networks (RMSE) summed up for the training and test data set at convergence of the training procedure. Additionally, the coefficient of determination (*r^2^*), the *y*-axis intercept, and the slope (*k*) of the linear least-squares regression analysis between observed and predicted values computed for the combined training and test data set as well as for the spatial data set are shown.
(PDF)

Table S4 **Radial basis function artificial neural network** (**RBF**)**-based models of the abundance of LNA cells.** The table gives the input parameters, the number of basis functions, and the root-mean-squared error of the networks (RMSE) summed up for the training and test data set at convergence of the training procedure. Additionally, the coefficient of determination (*r^2^*), the *y*-axis intercept, and the slope (*k*) of the linear least-squares regression analysis between observed and predicted values computed for the combined training and test data set as well as for the spatial data set are shown.
(PDF)

Table S5 **Feed-forward artificial neural network** (**FFW**)**-based models of the abundance of V1 viruses.** The table gives the input parameters, the number of hidden units, and the root-mean-squared error of the networks (RMSE) summed up for the training and test data set at convergence of the training procedure. Additionally, the coefficient of determination (*r^2^*), the *y*-axis intercept, and the slope (*k*) of the linear least-squares regression analysis between observed and predicted values computed for the combined training and test data set as well as for the spatial data set are shown.
(PDF)

Table S6 **Feed-forward artificial neural network** (**FFW**)**-based models of the abundance of V2 viruses.** The table gives the input parameters, the number of hidden units, and the root-mean-squared error of the networks (RMSE) summed up for the training and test data set at convergence of the training procedure. Additionally, the coefficient of determination (*r^2^*), the *y*-axis intercept, and the slope (*k*) of the linear least-squares regression analysis between observed and predicted values computed for the combined training and test data set as well as for the spatial data set are shown.
(PDF)

Table S7 **Radial basis function artificial neural network** (**RBF**)**-based models of the abundance of V1 viruses.** The table gives the input parameters, the number of basis functions, and the root-mean-squared error of the networks (RMSE) summed up for the training and test data set at convergence of the training procedure. Additionally, the coefficient of determination (*r^2^*), the *y*-axis intercept, and the slope (*k*) of the linear least-squares regression analysis between observed and predicted values computed for the combined training and test data set as well as for the spatial data set are shown.
(PDF)

Table S8 **Radial basis function artificial neural network** (**RBF**)**-based models of the abundance of V2 viruses.** The table gives the input parameters, the number of basis functions, and the root-mean-squared error of the networks (RMSE) summed up for the training and test data set at convergence of the training procedure. Additionally, the coefficient of determination (*r^2^*), the *y*-axis intercept, and the slope (*k*) of the linear least-squares regression analysis between observed and predicted values computed for the combined training and test data set as well as for the spatial data set are shown.
(PDF)

Text S1 **A short introduction to Artificial Neural Networks** (**ANNs**)**.**
(PDF)
