# Supplementary figures and images for: Modeling the Winter–to–Summer Transition of Prokaryotic and Viral Abundance in the Arctic Ocean
Source: PLoS One. 2012 Dec 20;7(12):e52794. doi: 10.1371/journal.pone.0052794 (PMC3527615; doi:10.1371/journal.pone.0052794)

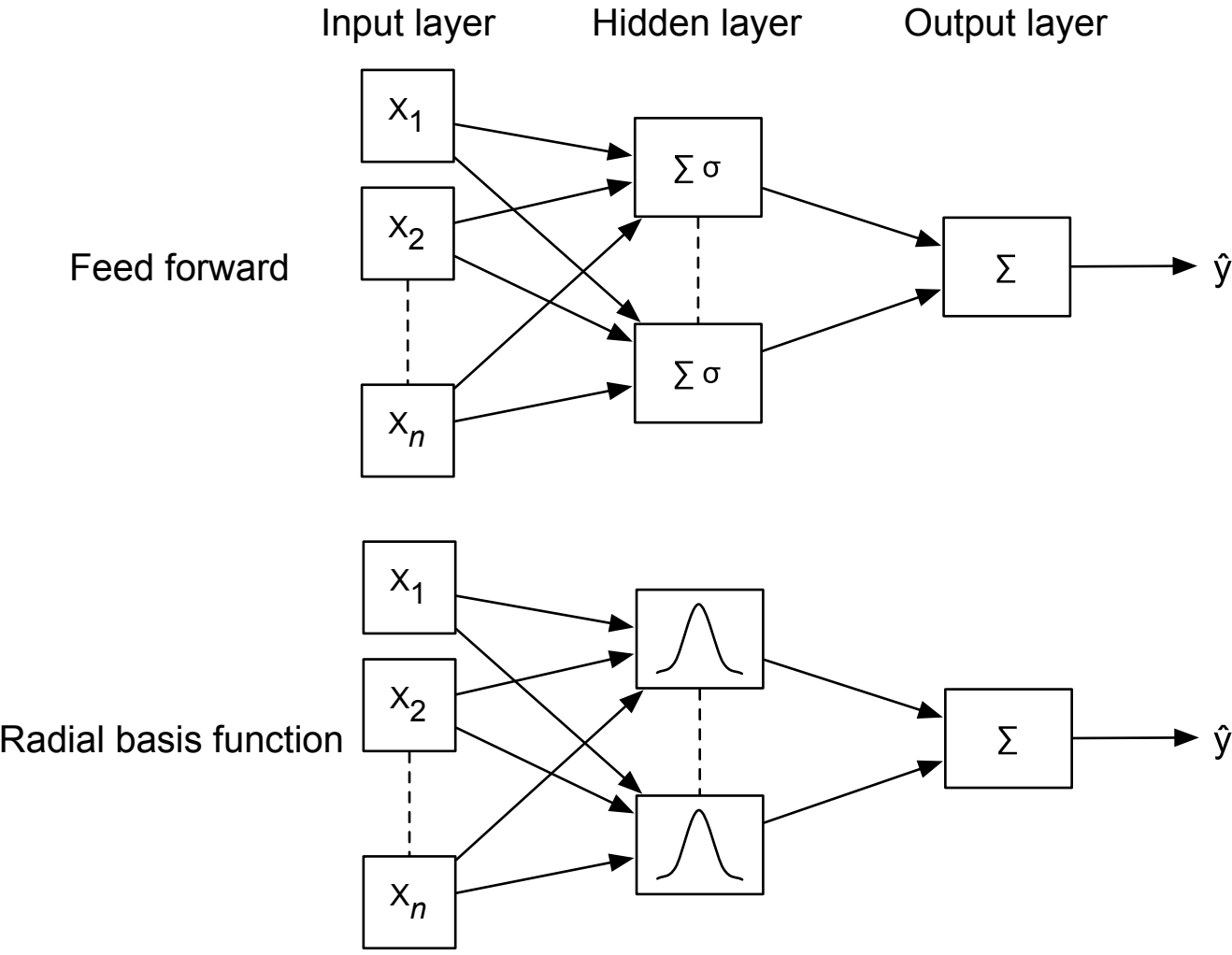

Figure S1  
Winter et al.

Supplement: Figure S1 — Schematic description of Artificial Neural Networks (ANNs). The figure details the network architectures of feed-forward (FFW) and radial basis function (RBF) ANNs used in this study. Data is fed into the input units (x1...xn) and transmitted along the weights to the hidden layer. The activation function for hidden units of FFW ANNs was the sigmoid function (σ) and for RBF ANNs the gaussian function was used. The output of the ANNs (ŷ) is compared to the known target values (y) and the difference is computed as the root-mean-squared error (RMSE). Bias terms are omitted for simplicity. (PDF) [file pone.0052794.s001.pdf]
