# Supplementary material for: Modeling the Winter–to–Summer Transition of Prokaryotic and Viral Abundance in the Arctic Ocean
Source: PLoS One. 2012 Dec 20;7(12):e52794. doi: 10.1371/journal.pone.0052794 (PMC3527615; doi:10.1371/journal.pone.0052794)
Supplement: Table S1 — Feed-forward artificial neural network (FFW)-based models of the abundance of HNA cells. The table gives the input parameters, the number of hidden units, and the root-mean-squared error of the networks (RMSE) summed up for the training and test data set at convergence of the training procedure. Additionally, the coefficient of determination (r2), the y-axis intercept, and the slope (k) of the linear least-squares regression analysis between observed and predicted values computed for the combined training and test data set as well as for the spatial data set are shown. (PDF) [file pone.0052794.s002.pdf]

Input parameters	Hidden units	RMSE	r^2	r^2-spatial	Intercept	Intercept-spatial	k	k-spatial
Chl-*a*, daylength	12	0.817	0.850	0.022	0.371	4.473	0.836	0.166
Chl-*a*, depth	8	0.763	0.851	0.001	0.350	4.731	0.835	-0.038
Chl-*a*, salinity	10	0.768	0.854	0.568	0.323	-3.837	0.864	2.204
Chl-*a*, temperature	14	0.706	0.901	0.559	0.169	-0.001	0.929	1.213
Chl-*a*, day length, depth	8	0.538	0.941	0.170	0.125	3.949	0.957	1.530
Chl-*a*, day length, sal.	8	0.549	0.931	0.138	0.152	4.216	0.910	0.643
Chl-*a*, day length, temp.	12	0.590	0.924	0.336	0.144	4.000	0.934	0.503
