# Supplementary material for: Modeling the Winter–to–Summer Transition of Prokaryotic and Viral Abundance in the Arctic Ocean
Source: PLoS One. 2012 Dec 20;7(12):e52794. doi: 10.1371/journal.pone.0052794 (PMC3527615; doi:10.1371/journal.pone.0052794)
Supplement: Table S2 — Feed-forward artificial neural network (FFW)-based models of the abundance of LNA cells. The table gives the input parameters, the number of hidden units, and the root-mean-squared error of the networks (RMSE) summed up for the training and test data set at convergence of the training procedure. Additionally, the coefficient of determination (r2), the y-axis intercept, and the slope (k) of the linear least-squares regression analysis between observed and predicted values computed for the combined training and test data set as well as for the spatial data set are shown. (PDF) [file pone.0052794.s003.pdf]

Input parameters	Hidden units	RMSE	r^2	r^2-spatial	Intercept	Intercept-spatial	k	k-spatial
Chl-*a*, daylength	13	0.790	0.864	0.440	0.280	1.594	0.868	0.772
Chl-*a*, depth	8	0.862	0.793	0.380	0.429	0.273	0.799	1.001
Chl-*a*, salinity	13	0.785	0.828	0.426	0.348	-2.370	0.830	2.274
Chl-*a*, temperature	13	0.769	0.866	0.196	0.339	2.459	0.836	0.430
Chl-*a*, day length, depth	14	0.686	0.905	0.150	0.238	1.798	0.894	0.555
Chl-*a*, day length, sal.	13	0.606	0.928	0.188	0.139	3.270	0.921	1.013
Chl-*a*, day length, temp.	15	0.723	0.742	0.464	0.472	3.136	0.808	0.369
