# Supplementary material for: Modeling the Winter–to–Summer Transition of Prokaryotic and Viral Abundance in the Arctic Ocean
Source: PLoS One. 2012 Dec 20;7(12):e52794. doi: 10.1371/journal.pone.0052794 (PMC3527615; doi:10.1371/journal.pone.0052794)
Supplement: Table S3 — Radial basis function artificial neural network (RBF)-based models of the abundance of HNA cells. The table gives the input parameters, the number of basis functions, and the root-mean-squared error of the networks (RMSE) summed up for the training and test data set at convergence of the training procedure. Additionally, the coefficient of determination (r2), the y-axis intercept, and the slope (k) of the linear least-squares regression analysis between observed and predicted values computed for the combined training and test data set as well as for the spatial data set are shown. (PDF) [file pone.0052794.s004.pdf]

Input parameters	Basis functions	RMSE	r^2	r^2-spatial	Intercept	Intercept-spatial	k	k-spatial
Chl-*a*, daylength	15	0.943	0.805	0.376	0.491	2.423	0.766	0.493
Chl-*a*, depth	12	0.926	0.789	0.624	0.532	-2.780	0.763	1.476
Chl-*a*, salinity	15	0.748	0.887	0.598	0.253	-3.777	0.895	1.742
Chl-*a*, temperature	15	0.713	0.879	0.606	0.261	-9.958	0.886	2.893
Chl-*a*, day length, depth	15	0.631	0.910	0.641	0.294	-6.182	0.884	2.010
Chl-*a*, day length, sal.	14	0.653	0.913	0.621	0.236	-2.923	0.886	1.735
Chl-*a*, day length, temp.	12	0.643	0.903	0.681	0.234	-8.215	0.889	2.690
