# Supplementary material for: Modeling the Winter–to–Summer Transition of Prokaryotic and Viral Abundance in the Arctic Ocean
Source: PLoS One. 2012 Dec 20;7(12):e52794. doi: 10.1371/journal.pone.0052794 (PMC3527615; doi:10.1371/journal.pone.0052794)
Supplement: Table S4 — Radial basis function artificial neural network (RBF)-based models of the abundance of LNA cells. The table gives the input parameters, the number of basis functions, and the root-mean-squared error of the networks (RMSE) summed up for the training and test data set at convergence of the training procedure. Additionally, the coefficient of determination (r2), the y-axis intercept, and the slope (k) of the linear least-squares regression analysis between observed and predicted values computed for the combined training and test data set as well as for the spatial data set are shown. (PDF) [file pone.0052794.s005.pdf]

Input parameters	Basis functions	RMSE	r^2	r^2-spatial	Intercept	Intercept-spatial	k	k-spatial
Chl-*a*, daylength	14	0.941	0.805	0.438	0.478	-2.378	0.766	1.790
Chl-*a*, depth	9	1.053	0.712	0.432	0.582	-0.912	0.694	1.255
Chl-*a*, salinity	12	0.967	0.801	0.459	0.462	-0.512	0.775	1.105
Chl-*a*, temperature	15	0.808	0.843	0.516	0.357	1.294	0.826	0.824
Chl-*a*, day length, depth	11	0.763	0.845	0.501	0.410	-1.517	0.803	1.220
Chl-*a*, day length, sal.	15	0.857	0.846	0.447	0.362	1.280	0.817	0.955
Chl-*a*, day length, temp.	9	0.747	0.854	0.503	0.316	-4.103	0.850	2.493
