# Supplementary material for: Modeling the Winter–to–Summer Transition of Prokaryotic and Viral Abundance in the Arctic Ocean
Source: PLoS One. 2012 Dec 20;7(12):e52794. doi: 10.1371/journal.pone.0052794 (PMC3527615; doi:10.1371/journal.pone.0052794)
Supplement: Table S5 — Feed-forward artificial neural network (FFW)-based models of the abundance of V1 viruses. The table gives the input parameters, the number of hidden units, and the root-mean-squared error of the networks (RMSE) summed up for the training and test data set at convergence of the training procedure. Additionally, the coefficient of determination (r2), the y-axis intercept, and the slope (k) of the linear least-squares regression analysis between observed and predicted values computed for the combined training and test data set as well as for the spatial data set are shown. (PDF) [file pone.0052794.s006.pdf]

Input parameters	Hidden units	RMSE	r^2	r^2-spatial	Intercept	Intercept-spatial	k	k-spatial
Chl-*a*, daylength	11	0.920	0.831	0.367	0.162	1.695	0.833	0.769
Chl-*a*, depth	11	0.676	0.912	0.078	0.053	1.653	0.972	0.617
Chl-*a*, salinity	10	0.809	0.870	0.059	0.110	2.264	0.886	0.524
Chl-*a*, temperature	8	0.863	0.848	0.000	0.134	1.327	0.855	0.009
Chl-*a*, day length, depth	15	0.453	0.962	0.229	0.025	1.149	0.979	0.910
Chl-*a*, day length, sal.	15	0.514	0.957	0.413	0.024	1.340	0.974	1.471
Chl-*a*, day length, temp.	11	0.583	0.938	0.370	0.044	0.863	0.959	1.129
