# Supplementary material for: Modeling the Winter–to–Summer Transition of Prokaryotic and Viral Abundance in the Arctic Ocean
Source: PLoS One. 2012 Dec 20;7(12):e52794. doi: 10.1371/journal.pone.0052794 (PMC3527615; doi:10.1371/journal.pone.0052794)
Supplement: Table S6 — Feed-forward artificial neural network (FFW)-based models of the abundance of V2 viruses. The table gives the input parameters, the number of hidden units, and the root-mean-squared error of the networks (RMSE) summed up for the training and test data set at convergence of the training procedure. Additionally, the coefficient of determination (r2), the y-axis intercept, and the slope (k) of the linear least-squares regression analysis between observed and predicted values computed for the combined training and test data set as well as for the spatial data set are shown. (PDF) [file pone.0052794.s007.pdf]

Input parameters	Hidden units	RMSE	r^2	r^2-spatial	Intercept	Intercept-spatial	k	k-spatial
Chl-*a*, daylength	14	0.857	0.828	0.196	0.853	7.479	0.842	0.243
Chl-*a*, depth	10	0.727	0.874	0.012	0.635	5.839	0.888	0.217
Chl-*a*, salinity	14	0.684	0.895	0.633	0.476	-9.403	0.904	1.932
Chl-*a*, temperature	11	0.798	0.856	0.089	0.774	8.043	0.855	0.270
Chl-*a*, day length, depth	8	0.669	0.905	0.544	0.455	3.728	0.915	0.781
Chl-*a*, day length, sal.	15	0.547	0.948	0.012	0.168	12.458	0.968	0.198
Chl-*a*, day length, temp.	13	0.643	0.912	0.099	0.400	9.286	0.921	0.285
