# Supplementary material for: Modeling the Winter–to–Summer Transition of Prokaryotic and Viral Abundance in the Arctic Ocean
Source: PLoS One. 2012 Dec 20;7(12):e52794. doi: 10.1371/journal.pone.0052794 (PMC3527615; doi:10.1371/journal.pone.0052794)
Supplement: Table S7 — Radial basis function artificial neural network (RBF)-based models of the abundance of V1 viruses. The table gives the input parameters, the number of basis functions, and the root-mean-squared error of the networks (RMSE) summed up for the training and test data set at convergence of the training procedure. Additionally, the coefficient of determination (r2), the y-axis intercept, and the slope (k) of the linear least-squares regression analysis between observed and predicted values computed for the combined training and test data set as well as for the spatial data set are shown. (PDF) [file pone.0052794.s008.pdf]

Input parameters	Basis functions	RMSE	r^2	r^2-spatial	Intercept	Intercept-spatial	k	k-spatial
Chl-*a*, daylength	15	0.910	0.846	0.551	0.151	0.235	0.844	2.049
Chl-*a*, depth	13	0.750	0.892	0.585	0.088	-0.150	0.917	1.838
Chl-*a*, salinity	14	0.747	0.898	0.484	0.100	0.831	0.891	0.728
Chl-*a*, temperature	14	0.887	0.850	0.609	0.119	-0.724	0.880	3.126
Chl-*a*, day length, depth	15	0.522	0.951	0.586	0.045	0.691	0.959	1.205
Chl-*a*, day length, sal.	14	0.625	0.933	0.632	0.053	0.182	0.945	1.803
Chl-*a*, day length, temp.	14	0.633	0.918	0.286	0.097	0.983	0.906	0.873
