# Supplementary material for: Modeling the Winter–to–Summer Transition of Prokaryotic and Viral Abundance in the Arctic Ocean
Source: PLoS One. 2012 Dec 20;7(12):e52794. doi: 10.1371/journal.pone.0052794 (PMC3527615; doi:10.1371/journal.pone.0052794)
Supplement: Table S8 — Radial basis function artificial neural network (RBF)-based models of the abundance of V2 viruses. The table gives the input parameters, the number of basis functions, and the root-mean-squared error of the networks (RMSE) summed up for the training and test data set at convergence of the training procedure. Additionally, the coefficient of determination (r2), the y-axis intercept, and the slope (k) of the linear least-squares regression analysis between observed and predicted values computed for the combined training and test data set as well as for the spatial data set are shown. (PDF) [file pone.0052794.s009.pdf]

Input parameters	Basis functions	RMSE	r^2	r^2-spatial	Intercept	Intercept-spatial	k	k-spatial
Chl-*a*, daylength	14	0.922	0.821	0.441	0.883	-10.005	0.825	2.371
Chl-*a*, depth	15	0.742	0.860	0.567	0.571	-5.563	0.888	1.499
Chl-*a*, salinity	7	0.817	0.854	0.059	0.694	3.812	0.867	0.319
Chl-*a*, temperature	10	0.805	0.848	0.467	0.727	-0.082	0.869	1.371
Chl-*a*, day length, depth	14	0.685	0.904	0.475	0.490	3.714	0.900	0.551
Chl-*a*, day length, sal.	15	0.603	0.934	0.503	0.249	5.245	0.950	0.673
Chl-*a*, day length, temp.	11	0.733	0.864	0.468	0.675	1.299	0.875	1.242
