# Supplementary material for: Modeling the Winter–to–Summer Transition of Prokaryotic and Viral Abundance in the Arctic Ocean
Source: PLoS One. 2012 Dec 20;7(12):e52794. doi: 10.1371/journal.pone.0052794 (PMC3527615; doi:10.1371/journal.pone.0052794)
Supplement: Text S1 — A short introduction to Artificial Neural Networks (ANNs). (PDF) [file pone.0052794.s010.pdf]

A short introduction to Artificial Neural Networks (ANNs)

The ANNs used in this study consisted of three layers of units (also called neurons), where each unit was fully connected to all units in the previous and the following layer (Fig. S1). The middle layer between input and output units is called the hidden layer. The connections between the units are called weights and can either enhance or weaken signals transmitted between connected units. During training, input data is presented to the network (the number of input units corresponds to the number of input parameters) and propagated along the weights to the units of the hidden layer. The principal difference between feed-forward (FFW) and radial basis function (RBF) ANNs lies in how the signals coming from the input layer are processed in the units of the hidden layer. For FFW ANNs, each hidden unit performs a weighted summation of the input signals, which then passes a nonlinear activation function, in this case the sigmoid function (σ). In the case of RBF ANNs, the numerical distance between the unit center and the input vector composed of the signals coming from the input units is calculated. The output of the hidden units is then formed by applying the basis function to this distance. For both, FFW and RBF ANNs, the network output is formed by another weighted summation of the outputs of the hidden units (the number of output units corresponds to the number of output parameters). At the output unit, the network output ($\hat{y}$) is compared to the known target values (y) and the difference between them is computed as the root-mean-squared error (RMSE). This error is then used further to decrease the networks error in the next iteration of the training process using the Levenberg-Marquardt algorithm [41,47] by adjusting the networks weights. Thus, during training the weights are iteratively adjusted to decrease the difference between the ANNs output and the known target values. For more detailed information on ANNs we refer to the review by Basheer and Hajmeer [42].
